# Investigating learning burnout and academic performance among management students: a longitudinal study in English courses

**DOI:** 10.1186/s40359-024-01725-6

**Published:** 2024-04-22

**Authors:** Thuy Dung Pham Thi, Nam Tien Duong

**Affiliations:** Department of Science, Technology and International Projects, Ho Chi Minh City University of Economics and Finance (UEF), 141-145 Dien Bien Phu, Ward 15, Binh Thanh District, Ho Chi Minh City, Vietnam

**Keywords:** Self-efficacy, Workload, Anxiety, Performance, Burnout

## Abstract

This study aims to move away from the cross-sectional approach related to burnout and conduct a longitudinal study to explore the factors influencing learning burnout among management students. The study primarily adopts a questionnaire survey, with students majoring in business management. Descriptive statistics and structural equation modeling (SEM) are used to analyze the data and validate the hypotheses. The findings are: (1) There is a significant negative relationship between English anxiety and self-efficacy and a significant positive relationship between past English learning performance and self-efficacy. (2) The changes in self-efficacy are negatively related to the changes in burnout, while the changes in workload are positively related to the changes in burnout. Additionally, there is a positive relationship between English anxiety and learning burnout. (3) There is a significant negative relationship between English learning performance and burnout. The direct impact of self-efficacy on English learning performance is not supported, but it has an indirect effect through the mediating role of burnout. The study proposes strategies to improve student outcomes and well-being: (1) making English courses more engaging to boost performance and confidence, reducing learning burnout; (2) encouraging and supporting students to enhance self-efficacy and motivation; (3) assigning tasks seen as useful and interesting to lessen perceived workload and emotional exhaustion; (4) and considering English anxiety in admissions to decrease learning burnout, especially as schools gain more autonomy in their policies.

## Introduction

Burnout has mainly focused on people-helping professionals who engage in long-term “people work” and experience a lack of enthusiasm toward their work, indifference towards people, and negative attitudes toward their job [1]. Previous studies on burnout have primarily targeted healthcare professionals, service industry employees, and social workers [2, 3]. Burnout among management professionals has been increasingly recognized as a significant issue, and studies have linked this phenomenon directly to management students, who represent a key future workforce in the field. Evidence suggests that burnout experienced during their academic years can be a precursor to similar challenges in their professional careers. This observation aligns with research indicating that burnout among students in professional programs, such as those preparing for careers in teaching, can predict future occupational burnout and affect job performance post-graduation. Pines and Kafry [4] comparing burnout levels between university students and professionals in people-oriented fields have shown that students often face higher burnout levels. This suggests that management students experiencing high burnout during their studies are at a greater risk of becoming professionals with significant burnout, affecting their productivity and job satisfaction. Therefore, identifying the factors that lead to burnout in management students is crucial. By understanding these factors, educators and institutions can better assess students’ academic performance and predict potential dropout risks, much like how assessing professional burnout can reveal employees’ job engagement or intentions to leave their positions.

Previous studies have shown that college students typically experience moderate to high levels of burnout. For example, Liu, Xie [5] found that over half of college students surveyed experienced academic burnout, with varying degrees of severity. This is further supported by Marôco, Assunção [6], who defined student burnout as exhaustion due to study demands, a cynical attitude towards schooling, and feelings of academic inefficacy. Further research suggests that burnout can occur when students perceive a lack of meaningful rewards or opportunities in their environment, leading to feelings of exhaustion and disengagement [7]. According to Lin and Kennette [8], students experiencing burnout often feel a lack of engagement and find classroom routines monotonous. Similar to employees in people-helping professions, student burnout is characterized by increased absenteeism, diminished motivation for coursework, and a higher likelihood of dropping out of college [9, 10]. Therefore, in this study, we define student burnout as a condition arising from academic stress, heavy course loads, or other psychological factors, leading to emotional exhaustion, a sense of depersonalization, and a diminished sense of personal achievement.

For the past years, in Vietnam, management colleges have required their students to simultaneously take both English-instructed management courses and English learning courses [11]. The integration of English-taught management courses aligns with the global trend of internationalization in higher education, where English is increasingly used as the medium of instruction in non-English speaking countries [12]. For students majoring in business management, the use of English in business management contexts, such as understanding international case studies, communicating in a global business environment, and reading and writing reports [13, 14], can lead to an increasingly common phenomenon of academic burnout during their studies. This phenomenon results in a lack of motivation, inability to concentrate on coursework, tardiness, early departures, and feelings of isolation. However, only a small portion of studies has found that academic burnout is an experience among college students, especially management-majored students [15, 16]. Therefore, this study attempts to conduct a longitudinal study to understand the factors that influence academic burnout among management students.

Studies point out that many students are afraid of English classes, lack confidence in themselves, experience anxiety, and doubt their abilities to handle the coursework [17]. Additionally, the top three sources of stress for students are academic pressure, lack of confidence, and feelings of loneliness [18]. As management students are potential future professionals in the field, some graduates who have worked hard to complete their management education end up not pursuing careers that are related to English. These graduates may have made such decisions due to feeling overwhelmed by academic pressures during their studies, fear of English, or a lack of confidence in their English. Consequently, they may actively avoid working in English or opt for non-English-related professions after graduation, resulting in a waste of educational resources.

Furthermore, numerous factors contributing to work-related burnout have been explored. These factors include environmental factors such as social/teacher support, digital competence, and workload [19, 20]; psychological factors such as grit, mindfulness, and emotion regulation [21, 22], as well as individual factors such as gender and personality traits [23–25]. However, most of these studies have focused on people-helping professionals [26, 27], medical students or college students, in general [28–30], with limited studies [31–33] longitudinally conducted on English learning burnout among college students, especially management students. Therefore, to cultivate better management talents, prevent the waste of educational resources, and provide valuable insights for teachers’ instruction, it is worth investigating the factors that contribute to academic burnout among management students.

Based on the aforementioned background and motivations, this study has two main objectives: (1) To understand the influence of intrinsic personal variables and external environmental factors on academic burnout, using a longitudinal study to investigate the impact of self-efficacy and academic workload on academic burnout. (2) To explore the effects of academic burnout and self-efficacy on English learning outcomes. In addition, the study will also investigate past English learning performance and the impact of English anxiety on self-efficacy among students in colleges of management in a non-native English-speaking country, Vietnam.

In conclusion, this study contribute to the understanding of English learning burnout, emphasizing the need to address this issue to enhance students’ learning experiences, well-being, and academic outcomes. The study offers valuable insights into the complex interplay of psychological, and educational factors contributing to burnout, highlighting the significance of addressing this issue in the context of English language education.

## Literature review

### Conservation of resources (COR) theory

The Conservation of Resources (COR) theory [34] serves as a foundational framework for understanding the origins of burnout and the responses associated with prolonged work-related stress. COR theory outlines the reasons certain situations are perceived as stressful and how individuals react to these stressful events. At its core, the COR theory posits that people are motivated to acquire and protect valued resources. Stress arises when there’s a threat to these resources, whether it is the potential for loss, actual loss, or even the pressure of significant gains. Specifically in the context of burnout, factors like physical exhaustion or excessive workload can undermine individuals’ confidence in their capacity to engage and sustain motivation, highlighting the COR theory’s emphasis on the impact of resource loss during times of stress.

Furthermore, anxiety can be viewed as both a result of resource threats and a contributing factor that exacerbates the perception of resource loss. It acts as a psychological mechanism that heightens the sensitivity to threats, amplifying the stress response and potentially accelerating the cycle of resource depletion.

### Student burnout

Leiter and Maslach [1] defined burnout as a phenomenon characterized by “emotional exhaustion,” “depersonalization,” and a diminished sense of personal accomplishment. Emotional exhaustion refers to a person’s lack of energy and feeling depleted of emotional resources, resulting in a lack of enthusiasm for work and often accompanied by feelings of frustration and tension. Depersonalization involves treating people as objects rather than individuals, exhibiting emotional detachment, callousness, cynicism, and a sense of estrangement towards clients, colleagues, or the organization. Diminished personal accomplishment refers to a person’s negative self-evaluation, feeling of inadequacy in their work, and a decrease in self-esteem.

Therefore, for students, this study adopts the definition of academic burnout as follows: “A phenomenon where students experience emotional exhaustion, depersonalization, and diminished personal accomplishment due to academic pressures, workload, or other personal psychological factors during their learning process.” This definition is based on Leiter and Maslach [1] and previous definitions of academic burnout [28, 35].

Indeed, many studies in the past have utilized the COR theory to explain the phenomenon of burnout [23, 34]. However, the findings from these studies have not been consistent. According to the COR, stress occurs when individuals perceive a threat to their valuable resources, experience a loss of resources, or fail to gain expected returns from investing their resources. For example, in the context of students, when they invest a significant amount of valuable time and energy into their academic coursework but do not achieve the expected outcomes, it can lead to feelings of stress. This aligns with the idea that the stress of burnout can arise when individuals perceive a loss or lack of resources, such as time, effort, or desired outcomes in their academic pursuits.

Within the COR, the workload is recognized as a significant environmental variable contributing to stress [36]. Workload refers to a situation where an individual faces multiple problems simultaneously within a limited time frame and is unable to resolve them, leading to a state of role overload [37]. The workload can impact a person’s physical health and job performance [38]. Past research has also found that academic workload is a primary factor contributing to student stress. Studies have shown that academic workload is consistently ranked as the top stressor among college students [39]. Villanova and Bownas [40] with 2,408 college students, the academic workload was identified as the most significant stress factor. Furthermore, factors such as exams, time pressure, and workload accounted for a substantial proportion (41.6%) of the perceived stress variance. Additionally, excessive stress has a negative impact on student learning [41]. Taken together, the aforementioned studies suggest that academic workload is one of the significant factors contributing to academic burnout among college students.

### Social cognitive theory and self-efficacy

Identifying and understanding the variations in individual behavior within different environments can often be challenging. Many traditional motivation theories that focus on cognitive processes and expectancies fall short in providing a detailed, process-oriented analysis of how individual actions influence environmental outcomes. Social Cognitive Theory (SCT) [42] addresses this gap by clearly defining the factors that determine human behavior. Self-efficacy is a central concept in the SCT that emphasizes how individuals assess their capabilities and make decisions regarding whether to engage in a particular behavior [43]. Social cognitive theory delves into the intricate interplay between (1) environmental influences like societal expectations and specific situations, (2) cognitive/personal factors encompassing individual traits and demographic information, and (3) behavior. Self-efficacy emerges as a vital mediator within this interaction, shaping behavior outcomes. Successful experiences boost individuals’ confidence in their abilities, enhancing their capability to navigate external circumstances.

Bandura [43] introduced a self-efficacy model, identifying four crucial determinants: (1) mastery experiences, stemming from past successes and seen as the most impactful on self-efficacy; (2) vicarious learning, where observing others’ successful actions helps adjust one’s self-efficacy; (3) verbal persuasion, using language or encouragement to boost self-efficacy; and (4) physiological and emotional arousal, affecting performance and self-efficacy perceptions.

Moreover, perceived self-efficacy significantly impacts various aspects of an individual’s life, including (1) choice behavior, influencing daily decisions on actions and time allocation; (2) performance and effort, where self-efficacy beliefs determine the dedication and perseverance applied to tasks; and (3) emotional reactions and thought patterns, with self-assessments of abilities shaping emotional responses and cognitive interactions with the environment.

### English anxiety

With rapid globalization, studying the psychological aspects of inner experiences among English learners has become increasingly important [44]. Anxiety is considered an important factor in learning English skills [45] because when students experience fear or anxiety towards English, it can affect their confidence, emotions, and behaviors related to English learning. Hashemi and Sciences [46] argue that anxiety is an emotion that is stable and can persist over a long period unless the learning environment is changed, such as increasing exposure to English courses or addressing the causes of anxiety. Changing a person’s beliefs and emotions (anxiety) requires a considerable amount of time [46]. Therefore, only by increasing the duration of English courses and providing more extended time to address learners’ English anxiety and attitudes, can anxiety be potentially changed. Thus, this study defines English anxiety as “an individual’s emotional fear, resistance, discomfort, or uneasiness towards English learning, accompanied by psychological discomfort or unease, which may potentially influence or hinder future English learning or perception of English”.

### Workload and burnout

In the context of the COR, the workload is considered an important environmental variable that contributes to stress [47]. It is also a significant factor in the demand aspect of the theory. When an individual’s valuable resources are threatened, both their physiological and psychological states can be affected. Workload refers to a situation where an individual faces numerous problems within a limited time frame and is unable to resolve them, resulting in role overload [37]. Excessive workload has been shown to impact both physical health and work quality [48]. Previous research has demonstrated associations between workload and outcomes such as burnout, increased cholesterol levels, excessive anxiety, and elevated heart rate [49]. Experiencing excessive workload not only affects employees’ health but also influences the way tasks are performed and employees’ perceptions of themselves and their work. Specifically, the excessive workload can lead to increased job dissatisfaction, reduced productivity with poor quality outcomes, burnout, anger, and feelings of personal failure [50].

The same situation can also occur in students. When students feel overwhelmed by the workload of school assignments within the available time, and they are unable to relieve the pressure or resolve academic problems, they can get caught in a downward spiral, exacerbating the situation. This can have an impact on students’ emotions and interpersonal relationships, leading to a loss of interest in schoolwork and a diminished sense of achievement. Leiter [51] proposed that burnout tends to worsen over time. In other words, individuals who initially experience burnout are likely to experience an increasing rate of burnout as time goes on. Rau, Gao [52] indicated that excessive academic pressure has a negative impact on college students’ learning. Previous studies pointed out that as students face an increasing course load or homework load, the occurrence of burnout significantly increases [53].

### Hypothesis development

The main purpose of this study is to investigate the factors influencing learning burnout among students in a management program based on the environmental factors of the COR and the individual factors of the SCT. Additionally, the study aims to understand the impact of learning burnout on English learning performance. This study adopts a longitudinal study design, which involves twice within one year to observe the learning burnout among the same group of students.

**Fig. 1:**
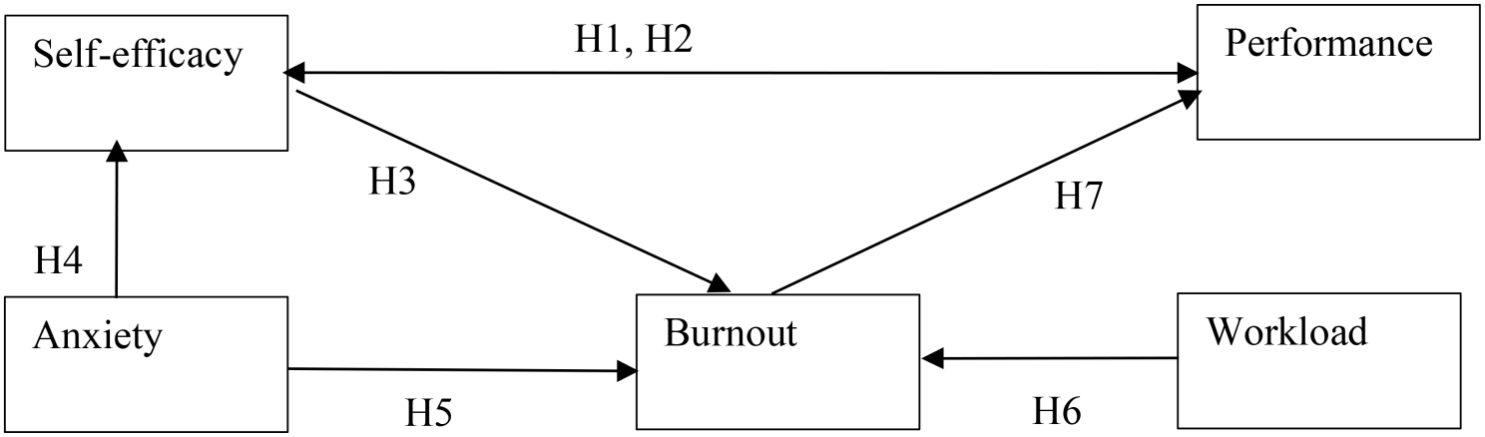
Conceptual model

The research model of this study, as shown in Fig. 1, combines the COR [34] and the SCT [43]. Based on the literature reviewed, we find that the factors influencing burnout are complex and cannot be explained solely by environmental stimuli. Previous studies on burnout among students have placed too much emphasis on environmental factors while not focusing on individual factors [10]. Additionally, burnout research should simultaneously consider the influence of individual and environmental factors [54], with self-efficacy being an important individual factor. However, can a student’s self-efficacy never change? If this self-efficacy does change, would it have an impact on their burnout over time? Previous self-efficacy research has mostly focused on cross-sectional studies, examining specific time points, and lacked longitudinal studies, which involve continuous time periods. This study aims to explore the impact of changes in self-efficacy on changes in learning burnout from a longitudinal perspective. Therefore, we hypothesize that when a student’s self-efficacy changes over time, their learning burnout will also vary. Several researchers have supported this argument through longitudinal studies, suggesting a positive relationship between self-efficacy at Time 1 and Time 2 [55, 56]. In other words, self-efficacy tends to increase or decrease over time. Additionally, those who experienced burnout in the previous phase had even higher levels of burnout in the subsequent phase [51]. This implies that burnout tends to increase over time. Thus, burnout is likely to worsen or lighten over time, and changes in self-efficacy can influence the trajectory of burnout.

Furthermore, an individual’s self-efficacy can influence their behavior [43]. When individuals perceive themselves as having higher self-efficacy, they are more likely to engage in social activism and cope with specific situations, making them less prone to psychological withdrawal. Rahmati and Sciences [57] argued that self-efficacy and burnout have a negative relationship. Therefore, considering that both self-efficacy and burnout can change over time, we can infer that the extent of self-efficacy change will affect the extent of burnout change [43]. In addition, previous studies have found that English anxiety can impact a person’s learning behavior and performance [58]. This suggests that in addition to environmental factors, individual differences are also important in influencing burnout [50]. Therefore, this study specifically examines the impact of students’ self-efficacy and English anxiety on learning burnout. Moreover, previous studies have identified academic workload as the primary source of stress for students [39]. Hence, in this study, the environmental variable focuses only on the workload. The excessive workload can lead to job dissatisfaction, slightly increased productivity but poor quality performance, feelings of stress, anger, and personal failure [50]. Research conducted in healthcare settings has also shown that the frequency of interactions with patients and the length of rest breaks can influence burnout. Additionally, the study includes the variable of students’ English learning performance since students typically care about their academic achievements.

The research model (Fig. 1) illustrates the relationships among the variables in this study. According to the model: (1) past English learning performance affects self-efficacy; English anxiety also influences self-efficacy; (2) self-efficacy affects learning burnout, and the extent of self-efficacy change influences the extent of learning burnout change; workload affects learning burnout, and the extent of workload change influences the extent of learning burnout change. Furthermore, English anxiety also affects learning burnout; (3) learning burnout influences current English learning performance, and self-efficacy influences current English learning performance. Based on the previous literature review and the research model, this study proposes the hypotheses.

Self-efficacy refers to an individual’s judgment of their capabilities to effectively accomplish their desired goals and tasks [59]. Based on the previous literature, self-efficacy is influenced by four main factors: performance accomplishments, vicarious learning, verbal persuasion, and physiological states [43]. Among these factors, performance accomplishments have been identified as the most important in shaping self-efficacy. Past successful experiences reinforce an individual’s belief in their abilities and increase their confidence. Conversely, repeated failures can lower self-efficacy. In other words, positive mastery experiences or achievements enhance self-efficacy, while negative experiences or failures can diminish them [60].

Empirical studies related to self-efficacy support the existence of the aforementioned relationship. Individuals with high self-efficacy tend to perform better than those with low self-efficacy [56]. Additionally, a meta-analysis on self-efficacy also confirmed the positive relationship between past performance and self-efficacy [61]. Furthermore, previous studies have found a positive correlation between students’ academic achievement and self-efficacy [62]. In summary, individuals with better academic performance tend to have higher self-efficacy, while those with poorer performance have lower self-efficacy. Therefore, we propose:

H1: Students with better past English learning performance will have higher self-efficacy, while students with poorer past English learning performance will have lower self-efficacy.

In Bandura and Adams [59], self-efficacy has an impact on outcomes, including one’s effort or performance. Self-efficacy influences an individual’s judgment to determine how much effort or persistence they will invest in completing a task or performing in a certain way. Individuals with higher self-efficacy are more energetic and persistent in their efforts to accomplish a task [63]. Especially when faced with difficult problems or tasks, individuals with lower self-efficacy may start doubting their abilities and weaken their efforts or even give up altogether, avoiding the challenges. On the other hand, individuals with stronger self-efficacy tend to exert more effort in overcoming or solving the difficulties and challenges they encounter [64].

Previous empirical research has extensively explored the relationship between self-efficacy and various outcomes, such as learning performance, job performance, and career decision-making. The results consistently indicate that individuals with higher self-efficacy tend to outperform those with lower self-efficacy in different domains. Studies have shown that individuals with higher self-efficacy exhibit better academic achievements [56], skill acquisition, performance in English learning or work effectiveness [65], and even career decision-making [66]. Based on these empirical findings, individuals with higher self-efficacy consistently exhibit better performance in various domains compared to those with lower self-efficacy. Therefore, we propose:

H2: Students with higher self-efficacy will have better English learning performance, while students with lower self-efficacy will have poorer English learning performance.

Bandura and Adams [59] stated that individuals with high self-efficacy have more confidence in themselves, while those with low self-efficacy have less confidence. An individual’s self-efficacy expectations can influence their behavior, which in turn affects their performance [59]. An individual’s self-efficacy will influence their emotional and cognitive responses. Individuals with high self-efficacy tend to have more positive emotional responses, while individuals with low self-efficacy generally have more negative emotional responses. When individuals complete challenging tasks on their own, it reinforces their work motivation and satisfaction [67]. This achievement leads to psychological success and encourages individuals to actively engage in their work. Conversely, it can lead to psychological withdrawal. Psychological failure can cause individuals to emotionally withdraw from the work environment, lower their work standards, and become indifferent [60, 68]. Therefore, based on this reasoning, it can be inferred that if students’ self-efficacy increases, their level of burnout should decrease, whereas if their self-efficacy decreases, their level of burnout should increase.

Students’ self-efficacy can indeed change over time [56]. Self-efficacy is not a fixed trait and can be influenced by various factors such as experiences, achievements, and feedback. If a student’s self-efficacy changes, it is reasonable to expect that it may have an impact on their level of burnout. In the past, self-efficacy research has mostly focused on cross-sectional studies, examining self-efficacy at specific time points, and lacking longitudinal studies that observe changes over time. However, our study aims to investigate the influence of changes in self-efficacy on changes in learning burnout using a longitudinal design, which is commendable. There is supporting evidence from longitudinal studies that self-efficacy can change over time. Researchers have found a positive relationship between self-efficacy at Time 1 and self-efficacy at Time 2 [55]. Additionally, Leiter [51] found that employees who had a burnout in the first stage experienced increased burnout in the second stage. These findings suggest that burnout can increase over time. Based on the understanding that both self-efficacy and burnout can change over time, it is reasonable to infer that the magnitude of self-efficacy change will influence the magnitude of burnout change. We can hypothesize that.

H3-1: Students with higher self-efficacy will experience lower levels of learning burnout, while students with lower self-efficacy will experience higher levels of learning burnout.

H3-2: Students who experience a greater increase in self-efficacy will have a larger reduction in the magnitude of learning burnout, whereas students who experience a greater decrease in self-efficacy will have a larger increase in the magnitude of learning burnout.

In addition to the influence of past English learning performance on self-efficacy, students’ self-efficacy may be influenced by other factors. According to Bandura [43], a person’s physiological or emotional arousal can impact their self-efficacy. Anxiety is a characteristic of physiological or emotional arousal. Kavanagh and Bower [69] proposed that emotions can influence a person’s self-efficacy, and individuals who experience feelings of depression often undermine their thoughts and abilities, perceiving a negative relationship between emotional arousal and self-efficacy. Agyapong, Obuobi-Donkor [70] suggested that psychological stress or anxiety hinders a person’s ability to discern the problems they face. Besides, individuals with higher English anxiety have lower self-efficacy, which consequently leads to lower English performance [45]. Additionally, many empirical studies have found that individuals with higher anxiety have lower confidence in themselves, while individuals with lower anxiety have higher confidence [59]. Liao, Wang [58] found that anxiety affects a person’s self-efficacy expectations, with individuals experiencing English anxiety having lower self-efficacy expectations. Therefore, we hypothesize:

H4: Students with lower levels of English anxiety will have higher self-efficacy, while students with higher levels of English anxiety will have lower self-efficacy.

Anxiety refers to the psychological responses of fear, discomfort, apprehension, or nervousness that an individual experiences toward certain events or situations [71]. These psychological responses often persist over time. Anxiety is considered an emotion that is believed to affect our attention to tasks and the processing of information we have learned [71]. Therefore, if students initially have psychological or emotional fear or aversion towards English, perceiving English as a threat, they are naturally inclined to reject them psychologically and lack the willingness to learn [46, 72]. They may find English unattractive and lack a sense of achievement. Endler and Kocovski [71] proposed that when individuals face psychological anxiety, they often adopt cognitive strategies of avoidance to ignore or deny the existence of the event. Therefore, if students have a preconceived fear of English, based on the aforementioned theoretical foundation, this English anxiety may affect their learning and possibly lead to the occurrence of learning burnout, resulting in strong feelings of frustration or low achievement in class. Indirectly, when students experience English anxiety, they may adopt a distant attitude towards English-related courses, skip classes, and feel a strong sense of frustration in learning English courses, leading to low performance and a lack of a sense of achievement. Past empirical studies indicated that English anxiety can affect students’ English performance [45]. Based on these findings, we can infer that.

H5: Students with higher levels of English anxiety will have higher levels of learning burnout, while students with lower levels of English anxiety will have lower levels of learning burnout.

In the structured model of burnout [50], it is evident that excessive workload is an important factor in job-related burnout. Furthermore, the COR argues that when individuals perceive a threat or loss of valuable resources, it can affect their mental and emotional well-being. The loss of resources and psychological distress are highly correlated [34]. Specifically, when individuals face an overwhelming number of tasks within the available time, it can lead to role overload, which has significant implications for the health and job quality of employees engaged in interpersonal work [37, 49]. Moreover, excessive workload can influence the way tasks are performed and how employees perceive their work. It often results in increased job dissatisfaction, decreased productivity with poor quality performance, mental strain, anger, and feelings of personal failure [47].

The same situation can also occur in students. When students feel overwhelmed by the excessive academic workload within the available time, unable to relieve the pressure or solve the academic challenges, they can spiral into a state of loss, exacerbating the situation [39]. This can affect students’ emotions and interpersonal relationships, leading to a loss of interest in schoolwork and a decrease in achievement satisfaction [73]. Besides, burnout tends to worsen over time, meaning that individuals who initially experience burnout are more likely to experience an increased rate of burnout [50]. Excessive academic pressure has negative effects on college students’ learning [39]. Previous studies have highlighted the close negative relationship between workload and burnout among students [74, 75]. Based on this, we infer that.

H6-1: The greater the academic workload of students, the more severe their learning burnout will be. Conversely, the lower the academic workload of students, the milder their learning burnout will be.

H6-2: Students who experience a larger increase in academic workload will have a greater increase in learning burnout. On the other hand, students who experience a larger decrease in academic workload will have a greater decrease in learning burnout.

In Shirom [76], emotional exhaustion was identified as a primary dimension of burnout. The intense emotional strain is predicted to interfere with effective functioning [1]. This perspective suggests a negative relationship between emotional exhaustion and performance. Therefore, for students, if they continuously experience an increasing burden or exhaustion in their emotions, may feel tired, depleted, irritable, frustrated, and emotionally drained, which can result in poor academic performance [77]. When students feel overwhelmed by their academic workload and unable to cope, they may exhibit behaviors of detachment, indifference towards classmates or school matters, and a lack of focus on their academic responsibilities, resulting in unsatisfactory academic performance. A reduced sense of accomplishment refers to a person’s perception of failure in terms of their abilities and work achievements [50]. Since burnout is considered a stress response, when students perceive a diminishing sense of accomplishment in their schoolwork, they may start to doubt their abilities, self-evaluate negatively, experience a sense of helplessness, and have diminished self-esteem, ultimately leading to poor learning outcomes [78, 79]. Individuals become less sensitive to others, exhibit negative emotions, and experience dissatisfaction after experiencing stressors, which leads to decreased job performance [80]. Anxiety and depression can impact job performance, with depression being considered one of the manifestations of burnout [70]. Paloș, Maricuţoiu [77] explored the relationship between student environment and academic burnout, finding a negative relationship between academic burnout and academic achievement. Studies found a negative relationship between emotional exhaustion and job performance, with emotional exhaustion being a key dimension of burnout [81, 82]. Based on these perspectives, we can infer that.

H7: Students with higher levels of academic burnout will have less satisfactory English learning performance, while students with lower levels of academic burnout will have more satisfactory English learning performance.

## Method

### Constructs and questionnaire design

In terms of operationalizing the research variables, reliable and valid scales from existing literature were utilized. The measurement of academic burnout adopted the Maslach Burnout Inventory-General Survey (MBI-GS) developed by Leiter and Maslach [1], as it is suitable for assessing academic burnout in the context of management students. The measurement of self-efficacy was based on Bandura [43], with modifications made to adapt it to the context of academic workload. The measurement of academic workload was adapted from scales developed by Kahn, Wolfe [37] that assess work demands. The measurement of English anxiety was based on the English anxiety developed by Tien [17] and Atef-Vahid, Kashani [45], which assesses individuals’ feelings of threat, fear, nervousness, unease, and hostile or resistant attitudes toward English during English learning. All the scale items above were measured by a 7-point scale.

In general, academic achievement refers to a student’s overall academic performance in school. However, in this study, the focus is solely on the grades of students in English-related subjects. The definition of learning achievement is based on Brown, Lent [83], which involves calculating the average grades of students in English subjects at the end of a semester. Specifically, there are two measures of English learning performance in this study. The first measure is the average of the grades in various English-related courses taken during the entire academic year of the first grade, referred to as “past English learning performance.” The second measure is the average of the grades in English-related courses taken during the entire academic year of the second grade, referred to as “prior English learning performance.” However, due to variations in English courses offered by the participating schools, the researchers first collected the course schedules of the schools. After analyzing and organizing the data, we found that the English courses offered by the participating schools followed a standardized curriculum. Therefore, the English learning achievement in this study primarily focused on standardized English courses. These courses involved both theoretical and practical components, and at the end of each semester, students were assigned a single grade for each course.

### Participants

This longitudinal study focused on management college students across Vietnam, selecting one to two schools from the northern, central, and southern regions for a representative sample. Due to the necessity for high cooperation from school teachers for administering two questionnaires, convenience sampling was employed, targeting more willing schools. A total of 11 classes from six management colleges were included. The first questionnaire was distributed two weeks prior to the first semester’s midterm exams in 2022 during class, and the second followed within two weeks after the second semester’s midterms, also conducted during class time.

In terms of data analysis, an exploratory factor analysis (EFA) was conducted to examine the discriminant validity of the variables’ scales. Principal Component Analysis (PCA) with varimax rotation was used to achieve orthogonal axes, and a significance level of item loading at 0.5 was adopted to determine the explanatory power of each variable scale [84]. Then, Cronbach’s alpha was computed to assess the reliability of each variable scale, with a cutoff value of 0.7 as the criterion for satisfactory reliability [85]. Descriptive statistics were utilized to observe the actual distribution of the sample. Additionally, academic performance was standardized. Since different schools have varying grading standards, with some being more lenient and others more strict, to make the grades more representative, the English grades were transformed into T-scores. The standardization process involved calculating Z-scores and then converting them into T-scores with a mean of 50 and a standard deviation of 10 [86]. Finally, a structural equation model (SEM) using path analysis was employed to test the hypotheses.

## Results

### Measurement of the constructs

A total of 615 questionnaires were collected for the first survey. After excluding 22 incomplete and invalid questionnaires, there were 593 valid responses, resulting in a response rate of 96.4%. Among the respondents, there were 154 males and 439 females. For the second survey, a total of 529 questionnaires were collected. After excluding 27 incomplete and invalid questionnaires, there were 502 valid responses for statistical analysis (considering those who completed both surveys), resulting in a response rate of 94.9%. Among the respondents, there were 120 males and 382 females.

First, the detection of common method bias issues was conducted using Harman’s single-factor test for exploratory factor analysis (EFA). The analysis revealed that 7 factors could be extracted, with the first factor explaining 37.807% of the variance and the sum of squared loadings for the rotation being 10.595%. Since this did not reach the 50% threshold for determining the presence of common method bias, the sample data does not have a severe issue with common method bias.

Next, an exploratory factor analysis (EFA) was conducted to examine the measurement model of the questionnaire. The “English burnout” measurement model was tested with EFA, utilizing principal component analysis to extract common factors, followed by the varimax method for orthogonal rotation based on extracted factors with Eigenvalues greater than 1. This process identified three factors: emotional exhaustion, depersonalization, and deminished personal accomplishment. The cumulative explained variance was 62.1%. For “English anxiety,” “workload,” and “self-efficacy,” the explained variances were 29.8%, 45.2%, and 55.5%, respectively.

To assess the reliability of the measurement instrument, an internal consistency (Cronbach α) test is conducted. For the current sample, the reliability analysis using Cronbach’s coefficient alpha was quite acceptable for overall burnout (α = 0.83), emotional exhaustion (α = 0.82), depersonalization (α = 0. 84), and diminished personal accomplishment (α = 0.76), anxiety (α = 0.81), self-efficacy (α = 0.78), workload (α = 0.84), suggesting that the questionnaire has relatively high reliability [85].

The study assessed the discriminant validity of its measurement model by examining the correlation coefficients and standard errors among different factors, ensuring that they are not equal to 1 within the sampling error range. Table 1 shows that all correlation coefficients between factors were below 0.5, confirming that the constructs are distinct and possess discriminant validity. For instance, the 0.24 correlation coefficient between English anxiety and learning burnout demonstrates their distinctiveness. Additionally, none of the correlation coefficients were 0, indicating substantive relationships among the factors. This further validates the measurement model used in the study [85].

**Table 1:** Correlations among the constructs

Constructs	Performance	Burnout	Anxiety	Workload	Self-efficacy
Performance	1				
Burnout	− 0.198***	1			
Anxiety	− 0.209***	0.240***	1		
Workload	− 0.053	0.398***	0.012	1	
Self-efficacy	− 0.305***	− 0.471***	− 0.356***	− 0.187***	1

*p < 0.05; **p < 0.1; ***p < 0.001

### Descriptive statistics

The variable distribution indicates slight variations in learning burnout and self-efficacy scores between two questionnaires, with average scores around 42.59/42.68 and 22.77/22.74, respectively. While differences are minimal, a slight decrease in self-efficacy is noted in the second questionnaire, hinting at a downtrend in students’ confidence in their abilities. Workload perception has increased, evident from average scores of 17.53 to 18.46. English anxiety, measured once due to its stability, showed an average score of 22.10 and an item mean of 2.21.

In this study, paired-sample t-tests were conducted to compare the differences between the pre-and post-periods for each variable. The results showed that there were no significant differences between the pre-and post-periods for learning burnout, self-efficacy, and English learning performance. However, there was a significant difference in the pre-and post-periods for the workload. The average score for workload in the post-period was higher than in the pre-period, indicating that students perceived an increasing workload. This difference in workload may be influenced by factors such as the duration of the college program and the arrangement of courses, which could explain the significant difference observed.

### Hypothesis testing

**Fig. 2:**
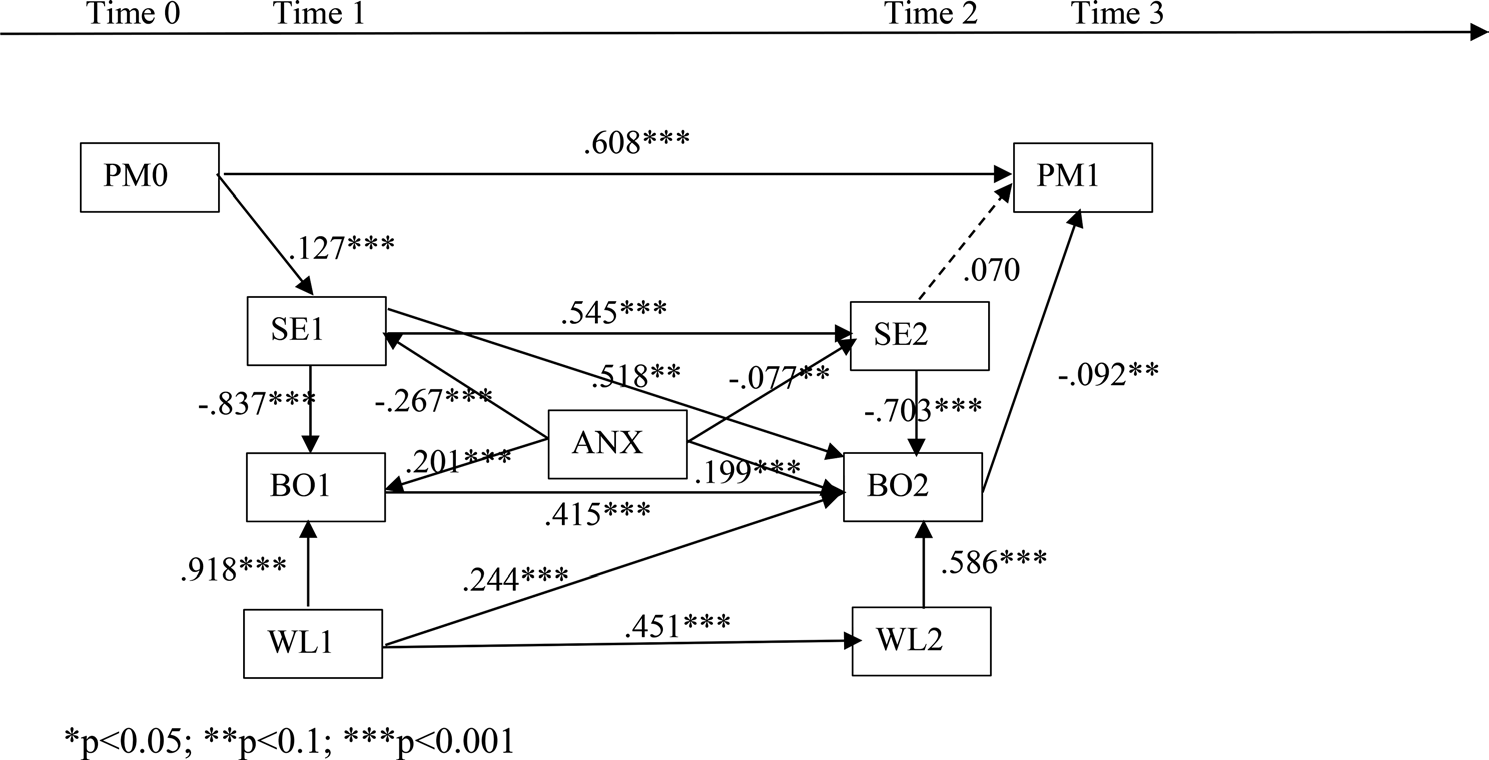
Path analysis. *p < 0.05; **p < 0.1; ***p < 0.001 *Note* PM = Performance; SE = Self-efficacy; BO = Burnout; ANX = Anxiety; WL = Workload

This study employed Structural Equation Modeling (SEM) to test the hypotheses (Fig. 2). “Performance 0” represents past English learning performance, " Performance 1” represents current English learning performance, " Self-efficacy 1” represents self-efficacy from the first questionnaire, " Self-efficacy 2” represents self-efficacy from the second questionnaire, “Burnout 1” represents learning burnout from the first questionnaire, " Burnout 2” represents learning burnout from the second questionnaire, “Workload 1” represents workload from the first questionnaire, “Workload 2” represents workload from the second questionnaire, and “Anxiety” represents English anxiety. There are 26 coefficients estimated in this study. To ensure an adequate sample size for SEM, the recommended guideline is a minimum of five times the number of estimated coefficients (Bentler & Chou, 1988). With 502 valid questionnaires in this study, the sample size meets the basic requirement for estimating the SEM. Therefore, a configuration of the limited information model is adopted. In the structural model, learning burnout is represented by the average sum of scores from the three dimensions: emotional exhaustion, depersonalization, and low personal accomplishment. The constructs of English anxiety, workload, and self-efficacy are also measured by the average sum of scores based on the limited information model configuration.

Furthermore, to meet the model identification requirement, this study sets the estimation parameters between factors and variables to 1. The estimation error variances of each variable are set to 1 minus the Cronbach’s α value of the corresponding construct, multiplied by the variance of that construct. This yields the estimation error variances (E) according to the formula: (1 - α) * σ^2^. In this study, the constructs that require the calculation of estimation error variances (E) in the SEM are “Self-efficacy 1”, " Self-efficacy 2”, “Burnout 1”, “Burnout 2”, “Anxiety”, “Workload 1”, and “Workload 2”. Additionally, for the English learning performance, as there is only one item with an average total score, the estimation error variances (E) for past English learning performance and current English learning performance are both set to 0 in this study.

Table 2 indicates that the relationship between self-efficacy and English learning performance is not significant (*p* > 0.1), while the remaining relationships are significant. The results from Table 2 are depicted in Fig. 2 to illustrate the path relationships in the structural model for the entire sample. In Fig. 2, the numbers on the paths represent the estimated path coefficients, and the asterisk (*) denotes the significance level of each path relationship. According to the model fit indices, CFI = 0.959 (> 0.9), NFI = 0.950 (> 0.9), NNFI = 0.902 (> 0.9), and AASR = 0.0467 (< 0.1), it can be concluded that the structural model demonstrates a good fit with the data.

**Table 2:** Results of path analysis

Construct		PM0	AXE1	BO1	BO2	SE1	SE2	WL1	WL2
SE1	Estimated Value SD t-value	0.127 0.022 5.833 ***	-267 036 -7.382 ***						
SE2	Estimated Value SD t-value		− 0.077 031 -2.500 **			0.545 0.035 15.432 ***			
BO1	Estimated Value SD t-value		0.201 0.076 2.633 ***			− 837 0.088 -9.543 ***		0.918 0.103 8.946 ***	
BO2	Estimated Value SD t-value		0.199 0.057 3.500 ***	0.415 0.033 12.582 ***		0.158 0.083 1.898 **	− 0.703 0.082 -8.528 ***	− 0.244 091 -2.672 ***	0.586 0.092 6.391 ***
PM1	Estimated Value SD t-value	608 036 17.118 ***			− 0.092 0.040 -2.296 **		070 0.081 0.862		
WL2	Estimated Value SD t-value							0.451 0.037 12.133 ***	

*p < 0.05; **p < 0.1; ***p < 0.001

*Note* PM = Performance; SE = Self-efficacy; BO = Burnout; ANX = Anxiety; WL = Workload

The complete SEM is supported by the path analysis. Therefore, based on the results of the path analysis and regression analysis mentioned earlier, the findings regarding the validation of each hypothesis are summarized in Table 3.

**Table 3:** Results of hypothesis testing

Hypothesis	Result
H1: Past English learning performance has a significant positive relationship with self-efficacy.	Supported
H2: Self-efficacy has a significant positive relationship with English learning performance.	Unsupported
H3-1: Self-efficacy (time 2) has a significant negative relationship with learning burnout (time 2).	Supported
H3-2: Self-efficacy (time 1) has a significant negative relationship with learning burnout (time 2).	Supported
H4: English anxiety has a significant negative relationship with self-efficacy.	Supported
H5: English anxiety has a significant positive relationship with learning burnout.	Supported
H6-1: Workload (time 2) has a significant positive relationship with learning burnout (time 2).	Supported
H6-2: Workload (time 1) has a significant positive relationship with learning burnout (time 2).	Supported
H7: Learning burnout has a significant negative relationship with English learning performance.	Supported

Finally, based on the standardized coefficients (Table 4), it is possible to determine the direct and indirect impacts of each variable on performance 1. This analysis allows for a comparison between the influence of internal and external variables on performance 1. When considering the cumulative effect of all variables on performance, PM0 emerges as the most significant predictor, followed by BO2, WL1, SE2, WL2, ANX, BO1, SE1. Therefore, it is evident that PM0 stand out as the primary determinants influencing performance 1.

**Table 4:** The effect of each variable on performance 1

Variables	Direct effect	Indirect effect	Total effect
PM0	0.60	0.127*0.518*(-0.092) + 0.127*(-0.837)*0.415*(-0.092) + 0.127*0.545*(-0.703)*(-0.092) = 0.002	0.610
SE1	None	0.518*(-0.092)+(-0.837)*0.415*(-0.092) + 0.545*(-0.703)*(-0.092) = 0.020	0.020
SE2	None	(-0.703)*(-0.092) = 0.06	0.060
BO1	None	0.415*(-0.092)=-0.04	-0.038
BO2	-0.092	None	-0.092
WL1	None	0.918*0.415*(-0.092) + 0.244*(-0.092) + 0.451*0.586*(-0.092)=-0.081	-0.081
WL2	None	0.586*(-0.092)=-0.053	-0.053
ANX	None	-0.267*0.545*(-0.703)*(-0.092)+(-0.077) *(-0.703)*(-0.092) + 0.201*0.415*(-0.092) + 0.199*(-0.092)=-0.040	-0.040

## Discussions

### Self-efficacy

Hypothesis 1 (H1): The impact of past English learning performance on self-efficacy. According to Bandura [43], an individual’s self-efficacy level is influenced by their past performance. Successful experiences strengthen one’s confidence, while unsuccessful experiences weaken it. This study found that students who performed well in English-related subjects in the past have higher self-confidence. On the other hand, students who had poor performance in English -related subjects in the past exhibit lower self-confidence.

Hypothesis 4 (H4): The impact of English anxiety on self-efficacy. According to Bandura [43], physiological or emotional arousal can influence an individual’s self-efficacy. A person’s bodily or emotional state can affect their judgment of confidence. Anxiety is considered one of the factors in this regard. This study found that students with higher levels of English anxiety tend to have lower confidence in their abilities related to English courses.

### Burnout

This study examines the impact of self-efficacy and workload on learning burnout based on social cognitive theory [43] and the COR [34], using a longitudinal research design. The hypotheses are described as follows:

H3-1 and H3-2: The influence of self-efficacy variability on learning burnout variability. Self-efficacy affects an individual’s emotional responses and cognitive patterns, and burnout is one of the emotional responses [43]. In other words, if individuals perceive that their self-efficacy is insufficient to meet the demands of the environment, it can affect their efforts and attention, leading to stress and impairing their judgment of their abilities, thereby resulting in emotional exhaustion or dehumanization. However, self-efficacy can change over time and can also influence changes in burnout [43, 57]. The present study found that fluctuations in self-efficacy negatively impact the extent of learning burnout fluctuations. This result suggests that self-efficacy may be strengthened or weakened through the accumulation of experiences over time, and the degree of this change is negatively related to the extent of learning burnout fluctuations.

H6-1 and H6-2: The influence of workload variability on learning burnout variability. According to the COR, when individuals perceive threats or losses to their valuable resources, it can affect their emotions or psychological well-being [34]. Therefore, when individuals feel that their workload is increasing, their experience of burnout tends to increase significantly. The present study found that as students’ academic workload increased over time, the extent of their learning burnout also increased noticeably [75]. Conversely, when students’ workload decreased, the extent of their learning burnout became less pronounced. Thus, fluctuations in workload directly and positively affect the fluctuations in students’ learning burnout.

H5: The influence of English anxiety on learning burnout. English anxiety refers to an emotional response at the psychological level toward English [17]. This study found that English anxiety has a positive impact on learning burnout. Students with higher levels of English anxiety experience more pronounced learning burnout. On the other hand, students with lower levels of learning burnout experience less pronounced learning burnout. Anxiety and burnout are positively related [70]; individuals with higher anxiety levels tend to exhibit impatience, indifference, reduced sense of accomplishment and decreased engagement in their tasks.

### Academic performance

H2: The influence of self-efficacy on English learning performance. An individual’s level of self-efficacy affects their performance and level of effort [43]. Individuals with higher self-confidence tend to perform better and are more willing to invest more effort into their work. Conversely, individuals with lower self-confidence tend to perform less effectively and are less willing to invest additional effort. The present study found that students’ performance in English learning is not necessarily better when their self-confidence is higher. Similarly, when students’ self-confidence is lower, their performance in English learning is not necessarily worse. The direct effect of self-efficacy on English learning performance is not significant, but English learning performance is influenced indirectly by the mediating effect of learning burnout. Therefore, the relationship between self-efficacy and English learning performance is mediated by learning burnout, which affects students’ English learning performance. The possible explanation for the result is due to the multifaceted nature of language acquisition and the critical role of psychological well-being in educational outcomes. English learning, being inherently complex, demands more than just high self-confidence; it requires consistent practice, exposure to the language, and effective learning strategies [87]. High self-efficacy might bolster the initial motivation and effort, but without addressing potential learning burnout, these efforts may not translate into improved performance. This suggests that burnout acts as a crucial mediator, with its potential to undermine the positive effects of self-efficacy by diminishing students’ motivation and capacity to engage with the learning material. Therefore, interventions aimed at enhancing English learning outcomes should not only foster self-confidence among learners but also create supportive learning environments that mitigate the risk of burnout.

H7: The influence of learning burnout on English learning performance. According to the COR, there is a negative relationship between emotional exhaustion, depersonalization, and job performance [34]. When an individual experiences burnout, their job performance tends to be less satisfactory. The present study found that as students’ learning burnout becomes more prominent, their performance in English learning becomes less satisfactory. There is a significant negative relationship between learning burnout and English learning performance. Therefore, it can be concluded that burnout affects an individual’s job performance.

### Implications

Based on these research findings, some recommendations can be made to the education field:

1. Enhance students’ English learning performance:

To effectively enhance students’ English learning performance, a strategic approach that combines interest-driven course design with supportive educational practices is essential. Initially, the study underscores the importance of designing English courses for freshmen that are simple, engaging, and tailored to capture their interest. Recognizing that students’ past performance in English impacts their self-confidence, which in turn influences learning burnout, prioritizing courses that spark interest from the outset encourages students to invest effort in learning. This approach not only improves English performance but also boosts self-confidence, thereby reducing the likelihood of burnout.

Building on this foundation, incorporating technology and multimedia, such as interactive applications and videos, caters to diverse learning styles and enhances the educational experience. Project-based learning, which addresses real-world challenges, along with customizable learning paths, further motivates students by highlighting the practical applications of English. Peer learning and discussion groups also create a collaborative environment that fosters communication skills and self-confidence. Additionally, establishing robust feedback mechanisms and support systems, including access to tutoring and counseling, ensures continuous improvement and provides necessary encouragement. Cultural and linguistic immersion experiences, such as cultural exchange programs or interactions with native speakers, significantly contribute to linguistic proficiency and cultural awareness.

(2) Enhance students’ self-efficacy:

To bolster students’ self-efficacy and counteract learning burnout, this study proposes a comprehensive strategy rooted in the positive correlation between self-efficacy and learning outcomes. Bandura’s theory highlights the impact of verbal persuasion and social support on self-confidence, suggesting that educators’ regular verbal encouragement and support play a critical role in motivating students. By fostering an affirming environment where students feel valued, their self-confidence is likely to increase, thereby enhancing their self-efficacy.

Therefore, the strategy encompasses several key elements to further support students. Personalized feedback is crucial, focusing on students’ efforts and progress rather than just the outcomes, thereby validating their individual learning journeys and emphasizing growth. Setting achievable goals allows students to experience incremental success, strengthening their belief in their own abilities. The inclusion of role models and the promotion of observational learning act as powerful motivators, illustrating that resilience can lead to success. Skill development workshops on practical skills like time management and stress management provide students with essential tools for academic success, augmenting their self-efficacy. Moreover, creating a supportive community through study groups or mentorship programs helps alleviate feelings of isolation, fostering a sense of belonging and mutual support. Celebrating students’ achievements, no matter the scale, plays a significant role in reinforcing their sense of competence and motivation.

(3) Reduce students’ perception of excessive workload and make English assignments useful and interesting:

To address the challenge of perceived excessive workload and its impact on learning burnout among students, a logical and strategic approach is necessary. Recognizing that heavy workload perceptions contribute to burnout, it’s essential for educators to adjust the way English assignments are structured and perceived. Assignments should be designed to be meaningful, engaging, and clearly connected to real-world applications, thereby enhancing their relevance and interest to students. This entails incorporating practical examples, interactive elements, and projects that allow students to apply what they learn in tangible ways. By making assignments more engaging and relevant, students are more likely to view them as valuable learning opportunities rather than burdensome tasks.

Further, integrating techniques such as gamification can make learning more interactive and enjoyable, thereby reducing the perception of workload. Project-based assignments that encourage deep exploration of topics not only make learning more interesting but also foster a deeper sense of accomplishment and ownership over the learning process. Allowing students some choice in their assignments can also personalize the learning experience, increasing engagement and reducing feelings of overload. Breaking down assignments into smaller, more manageable tasks with clear, achievable objectives can help students better manage their workload and reduce stress. Providing constructive, timely feedback and recognizing achievements can further motivate students and support a growth mindset.

(4) Considering English anxiety as a criterion for selecting students:

Acknowledging the impact of English anxiety on student performance, including self-confidence and susceptibility to learning burnout, there’s a clear need for educational institutions to refine their admission policies. English anxiety can manifest as impatience, indifference, and a diminished motivation for achievement, adversely affecting students’ academic progress. As educational institutions move towards gaining greater autonomy over their admission criteria, shifting from a sole reliance on standardized testing to more comprehensive evaluations offers a strategic opportunity to address these challenges.

Incorporating an assessment of English anxiety into the selection process can lead to several positive outcomes. First, it paves the way for a learning environment that’s less prone to inducing burnout, by selectively admitting students whose anxiety levels are within a manageable range. This proactive measure can substantially improve the learning atmosphere, making it more supportive and reducing stress for all students. Furthermore, identifying students with lower levels of English anxiety allows for a targeted approach to support, including the introduction of programs aimed at building confidence and alleviating anxiety, thus promoting better academic and personal well-being.

### Future research

The study outlines future research directions, emphasizing the need to broaden its scope beyond management program students and explore potential learning burnout impacts across various educational systems and college programs. It highlights the necessity of validating the study’s applicability to diverse student groups, given the comparable coursework pressures. Furthermore, the study calls for a critical evaluation of the research tools employed, as many were sourced internationally or from different fields, potentially introducing bias. Future work should prioritize creating culturally relevant and context-specific measurement tools to enhance the accuracy and reliability of research findings.

## Data Availability

The data that support the findings of this study are available from the corresponding author upon reasonable request.
